# Type I-F CRISPR-PAIR platform for multi-mode regulation to boost extracellular electron transfer in *Shewanella oneidensis*

**DOI:** 10.1016/j.isci.2022.104491

**Published:** 2022-05-30

**Authors:** Yaru Chen, Meijie Cheng, Hao Song, Yingxiu Cao

**Affiliations:** 1 Frontier Science Center for Synthetic Biology and Key Laboratory of Systems Bioengineering (Ministry of Education), School of Chemical Engineering and Technology, Tianjin University, Tianjin 300072, China; 2 Key Laboratory of Systems Bioengineering (Ministry of Education), Tianjin University, Tianjin 300072, China

**Keywords:** Biochemistry, Biochemical engineering, Metabolic engineering

## Abstract

Bio-electrochemical systems are based on extracellular electron transfer (EET), whose efficiency relates to the expression level of numerous genes. However, the lack of multi-functional tools for gene activation and repression hampers the enhancement of EET in electroactive microorganisms (EAMs). We thus develop a type I-F CRISPR/PaeCascade-RpoD-mediated activation and inhibition regulation (CRISPR-PAIR) platform in the model EAM, *Shewanella oneidensis* MR-1. Gene activation is achieved (3.8-fold) through fusing activator RpoD (σ^70^) to Cas7 when targeting the prioritized loci upstream of the transcription start site. Gene inhibition almost has no position preference when targeting the open reading frame, which makes the design of crRNAs easy and flexible. Then CRISPR-PAIR platform is applied to up-/down-regulate the expression of six endogenous genes, resulting in the improved EET efficiency. Moreover, simultaneous gene activation and inhibition are achieved in *S. oneidensis* MR-1. CRISPR-PAIR platform offers a programmable methodology for dual regulation, facilitating in-depth EET studies in *Shewanella* spp.

## Highlights

• CRISPR-PAIR platform enables both gene activation and inhibition in *Shewanella oneidensis*
• An efficient type I-F CRISPR-Cas tool is developed for *S. oneidensis*
• Transcription regulation of endogenous genes enhances extracellular electron transfer (EET)

## Graphical abstract

**Figure:**
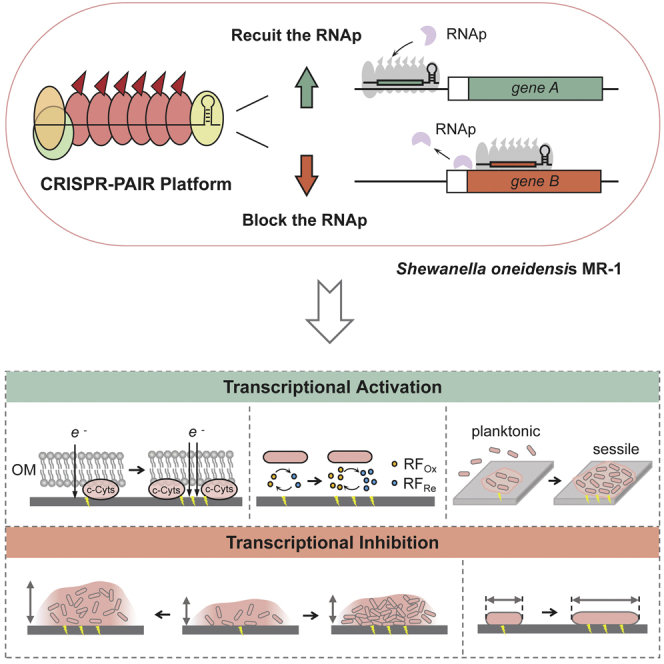

## Introduction

Electroactive microorganisms (EAMs) utilize the bidirectional extracellular electron transfer (EET) pathway to exchange electrons with the environment and enable a variety of microbial electrochemical techniques (METs) (Logan et al., 2019). *Shewanella oneidensis* MR-1 is regarded as an important model EAM; however, the relatively low EET efficiency of wild-type *S. oneidensis* MR-1 severely limits practical applications (Fredrickson et al., 2008; Logan et al., 2019; Shi et al., 2016), driving it necessary to conduct elaborate genetic engineering. The mechanism of EET is highly sophisticated, including direct EET pathways mainly mediated by outer-membrane cytochromes (OM-cyts) (Shi et al., 2007), and indirect EET pathways that function via self-secreted electron shuttles (Brutinel and Gralnick, 2012; Gralnick and Newman, 2007; Watanabe et al., 2009). In addition, complex metabolic networks and multiple cellular activities also affect the efficiency of EET, such as the anaerobic respiration pathway (Liu et al., 2017), biofilm formation (Sivakumar et al., 2014), endogenous electron shuttle (Mevers et al., 2019; Zou et al., 2017), and carbon source utilization (Li et al., 2017). Many genes associate with these complicated processes and are thus needed to be regulated, either up or down, to match the highly active electron transporting. Hence, multi-level modulation of gene expression is critical to promote EET and broaden the applications of METs in *S. oneidensis* MR-1 (Meitl et al., 2009).

To date, there have been various tools available to artificially carry out gene regulation. For enhancing gene expression, the current approaches, plasmid-based overexpression and genomic knock-in, are utilized to increase the expression level of the specific genes in *S. oneidensis* MR-1 (Fan et al., 2021a, 2021b; Min et al., 2017). For repressing gene expression, CRISPRi system efficiently blocks RNA polymerase binding or elongation to achieve transcriptional interference (Cao et al., 2017; Li et al., 2020). However, the above approaches of enhancing and repressing gene expression are two types of totally separate systems. These mono-functional systems could not easily meet the requirements for the modulation of substance and energy metabolism in *S. oneidensis* MR-1. Therefore, the development of a dual-regulation tool which enables both gene activation and gene inhibition is urgently needed, not only to reduce the time cost of mining the mechanism of EET, but to obtain more engineered strains with high EET efficiency readily.

**Figure 1:**
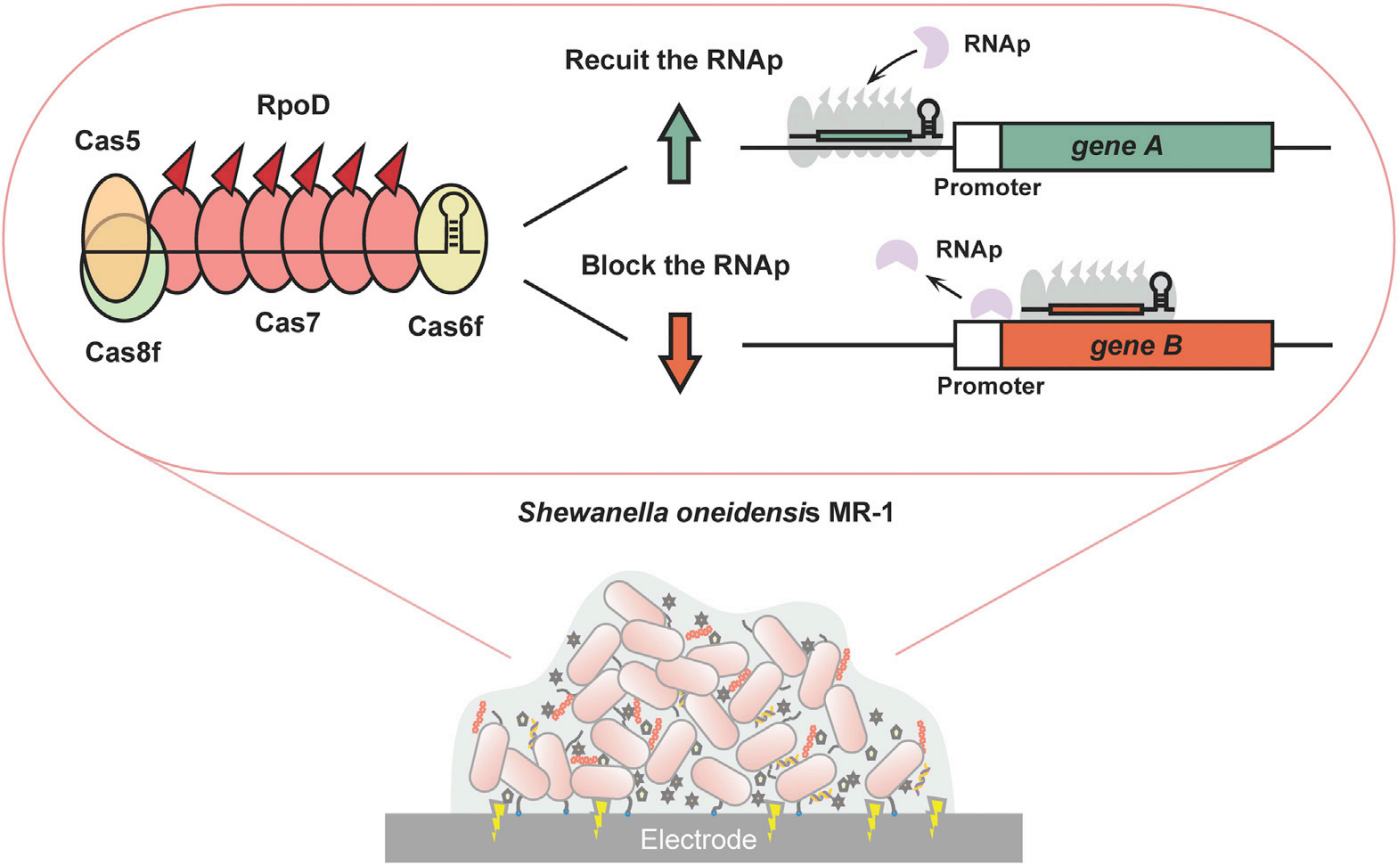
The programmable type I-F CRISPR/PaeCascade-RpoD-mediated activation and inhibition regulation (CRISPR-PAIR) platform developed in *S. oneidensis*

Type I and type II CRISPR-Cas systems have been widely used as the tool for gene manipulation (Chang et al., 2016; Chen et al., 2020; Lian et al., 2017). Despite pioneering work with type II CRISPR-Cas tools for different functions in diverse species (Li et al., 2015; Lian et al., 2017), type I CRISPR-Cas systems have many attractive features as a transcriptional regulation tool for *S. oneidensis* (Semenova et al., 2015; Zheng et al., 2019). Firstly, type I CRISPR-Cas systems are prevalent in the species close to *S. oneidensis,* such as *Shewanella putrefaciens* (Dwarakanath et al., 2015) and *Pseudomonas aeruginosa* (Xu et al., 2019), elevating the possibility of choosing an appropriate system which would function effectively in *S. oneidensis* MR-1. Secondly, type I system relies on a cascade for DNA binding and Cas3 for degrading the foreign DNA (Westra et al., 2012). The subunit responsible to bind the protospacer in type I cascade has several copies, unlike type II Cas protein functioning as a single molecule (Xu et al., 2019). In this case, more effectors for activation or inhibition are able to be recruited to this subunit (Figure 1) (Chen et al., 2020). These properties make type I CRISPR-Cas system an ideal two-way regulation tool for large-scale control of the electron flux in EAMs.

In this study, we developed a dual-regulation tool using type I CRISPR-Cas system in *S. oneidensis* MR-1 (Figure 1). Firstly, PaeCascade (Cascade of type I-F CRISPR-Cas system from *P. aeruginosa*) was selected from three candidates as a suitable system in *S. oneidensis* MR-1. Secondly, for gene activation, PaeCascade-RpoD was constructed by fusing activator RpoD with Cas7 (functioning as several copies to bind the protospacer). The prioritized targeting sites for activation were identified and 3.8-fold activation was successfully achieved via type I-F CRISPR-Cas system in *S. oneidensis* MR-1. For gene inhibition, there was almost no position dependence when targeting the open reading frame (ORF), thus it is quite flexible to design the crRNAs. Thirdly, we utilized PaeCascade-RpoD system to activate 3 genes and repress 3 genes, related to biofilm formation, outer-membrane cytochrome, and so forth. The EET efficiency of all regulated strains was improved, and corresponding phenotype changes including thicker biofilm and longer cell morphology were observed. Finally, the feasibility of simultaneous gene activation and inhibition was verified in *S. oneidensis* MR-1. In sum, type I-F CRISPR/PaeCascade-RpoD-mediated activation and inhibition regulation (namely CRISPR-PAIR) platform provides a programmable and facile methodology for dual modulation, which would facilitate comprehensive EET studies and multi-dimensional MET applications in *Shewanella* spp.

## Results

### To screen and characterize type I CRISPR-Cas systems in *S. oneidensis*

**Figure 2:**
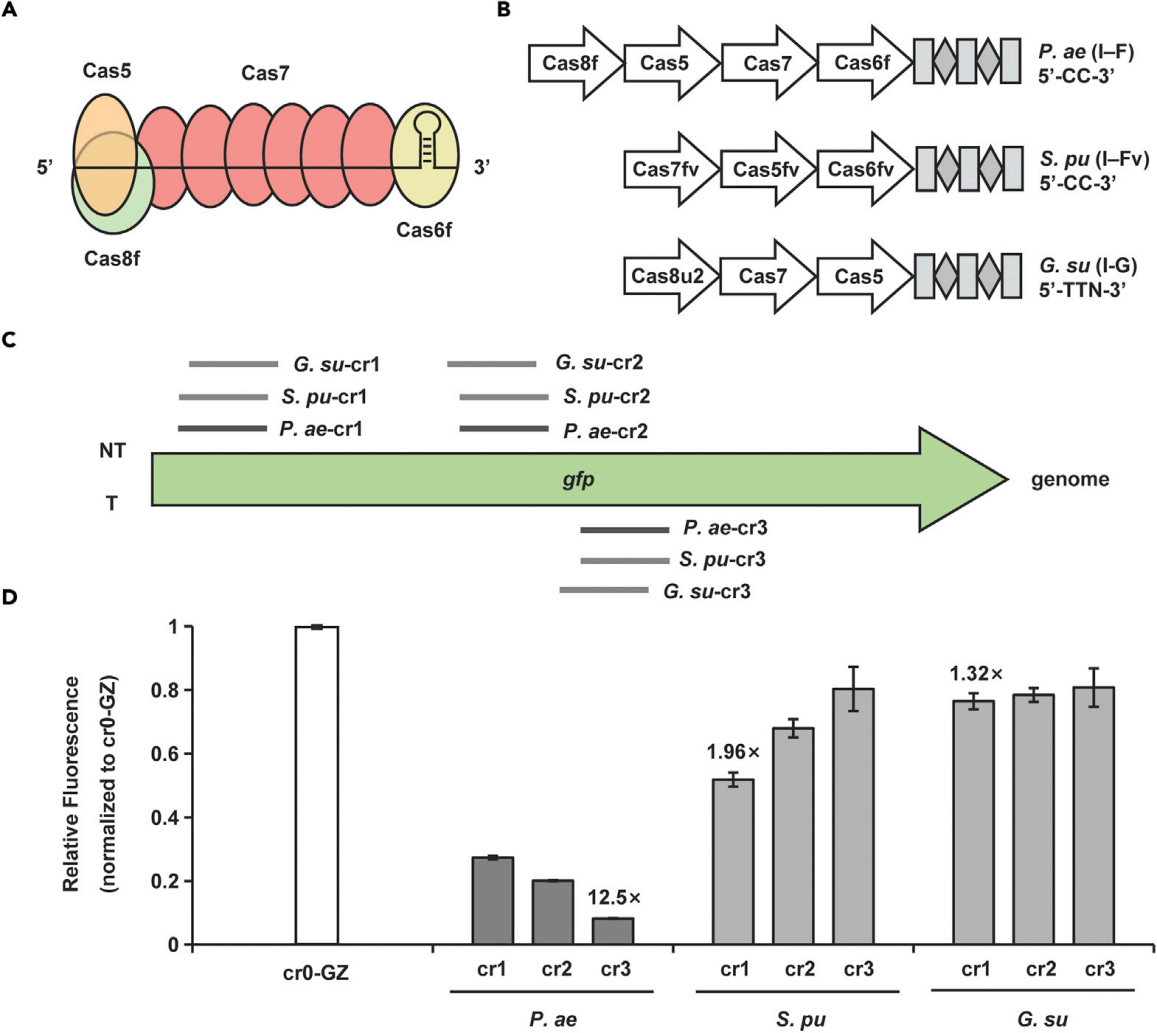
To screen type I CRISPR-Cas systems in *S. oneidensis* (A) The structure of PaeCascade-crRNA complex. Cas5 binds to the 5′ handle of the crRNA and Cas8f is responsible for PAM recognition (5′-CC-3′) at the 5′ end of the protospacer. Cas7 proteins function as several copies bind to the protospacer, serving as the backbone of the cascade complex. Cas6f combines with crRNA 3′ hairpin structure and mediates pre-crRNA maturation. (B) The schematic diagram of the *P. aeruginosa* type I-F (*P. ae* I-F), *S. putrefaciens* type I-Fv (*S. pu* I-Fv), and *G. sulfurreducens* type I-G (*G. su* I-G) CRISPR-Cas systems used in this study. Cas proteins are presented with arrows. CRISPR repeats and spacers are indicated with squares and diamonds, respectively. (C) Targeting sites of designed crRNAs binding different positions of *gfp* in the genome of the strain *S. oneidensis* GZ for three type I CRISPR-Cas systems. Three crRNAs were designed for each system. (D) The inhibition efficiency of three type I CRISPR-Cas systems targeting *gfp*. The values above the bar indicate the highest repression folds of three crRNAs for each system. Median GFP levels are normalized to the control strain cr0-GZ. Values and error bars indicate mean ± standard error of mean (SEM) of three replicates.

Type I CRISPR-Cas systems have the effector modules consisting of multiple Cas proteins which function together in binding and processing the target (Luo et al., 2015). Taking *Pseudomonas aeruginosa* type I-F CRISPR-Cas system (PaeCascade) as an example, PaeCascade complex is composed of 4 kinds of Cas protein subunits, Cas8f, Cas5, Cas7, and Cas6f (Chowdhury et al., 2017; Wiedenheft et al., 2011) (Figure 2A). Cas8f is responsible for recognizing PAM (5’-CC-3’) at the 5’ end of the protospacer and Cas5 binds to the 5’ handle of the crRNA. Cas6f combines with crRNA 3’ hairpin structure and mediates pre-crRNA maturation. Cas7 functioning as several copies binds to the protospacer, serving as the backbone of the cascade complex (Gleditzsch et al., 2016; Gu et al., 2019). Compared to the standalone dCas9, more than one effector for activation or inhibition fused to Cas7 can be recruited to the targets, allowing stronger transcriptional regulation (Figure 1) (Cady et al., 2012; Chen et al., 2020).

Several type I CRISPR-Cas systems have been discovered, driving it necessary to find a suitable one for application in *S. oneidensis* MR-1. We thus chose three candidates from close relative species of *S. oneidensis*, PaeCascade (*Pseudomonas aeruginosa* type I-F CRISPR-Cascade) (Luo et al., 2015), SpuCascade (*Shewanella putrefaciens* type I-F variant CRISPR-Cascade) (Gleditzsch et al., 2016) and GsuCascade (*Geobacteraceae sulfurreducens* type I-G CRISPR-Cascade) (Makarova et al., 2020) (Figure 2B).To determine the ability of transcriptional regulation of these three systems in *S. oneidensis*, we adopted *gfp* as a reporter in the genome (*S. oneidensis* GZ) (Li et al., 2020) for transcriptional interference. crRNAs (cr1, cr2, cr3) either targeting the template strand (T) or the non-template strand (NT) were designed to repress the expression of *gfp* (Figure 2C). As shown in Figure 2D, after 24-h incubation, the inhibition efficiency of PaeCascade for *gfp* expression was up to 12.5-fold in *S. oneidensis*. However, the highest repression efficiencies of SpuCascade and GsuCascade were only 1.96-and 1.32-fold, respectively. Such differences reaffirm the notion that different cascade complexes have distinct properties from one another (Zheng et al., 2020). Hence, in the following sections, we will focus on type I-F PaeCascade system and investigate how to utilize it efficiently to modulate transcriptional level.

**Figure 3:**
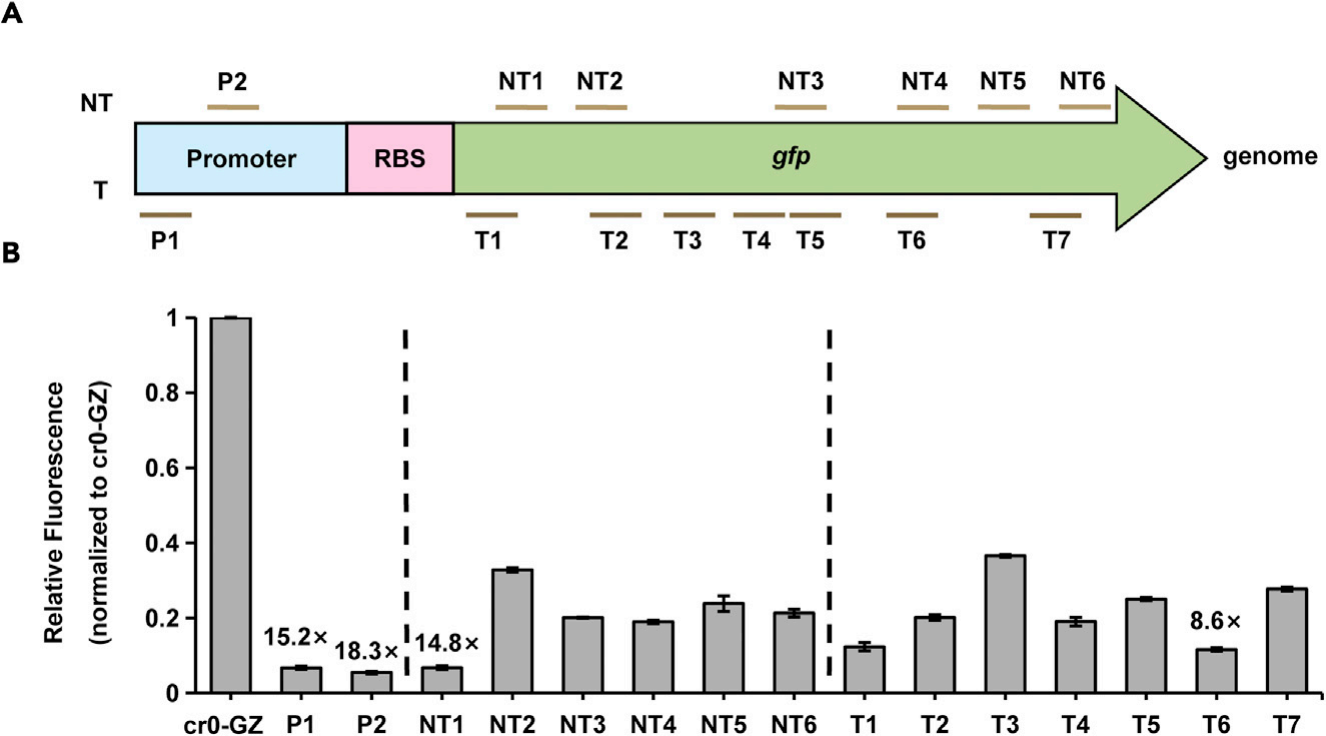
To characterize optimal targeting sites of PaeCascade-mediated CRISPRi in *S. oneidensis* (A) Targeting sites of designed crRNAs binding different regions of *gfp* in the genome of the strain *S. oneidensis* GZ by type I-F PaeCascade-mediated system. (B) The inhibition efficiency of type I-F PaeCascade system targeting different regions of *gfp*. The values above the bar indicate the corresponding repression folds. Median GFP levels are normalized to the control strain cr0-GZ. Values and error bars indicate mean ± SEM of three replicates.

We next identified the correlation between PaeCascade-mediated repression and the targeted sites. crRNAs were designed complementary to different regions of the *gfp* sequence (NT1∼NT6, and T1∼T7), or promoter (P1 and P2) (Figure 3A). Spacers of crRNA were constructed through the Golden Gate assembly method (Engler et al., 2008; Fang et al., 2021). The strains harboring the plasmid with PaeCascade and corresponding crRNA were incubated for 24 h, and the fluorescence intensities were detected. In comparison to the strain with spacer-free plasmid (the strain termed cr0 hereafter), strains targeting P1, P2 and NT1 demonstrated high repression efficiency, which was ∼15.2-fold, ∼18.3-fold, and ∼14.8-fold, respectively (Figure 3B). Besides, interference levels were similar when targeting other sites, from 2.5-fold to 5-fold, no matter if the targeting sites were adjacent to or far from the initiation codon, on template or non-template strand. (Figure 3B). The phenomena were different from what were observed in type II CRISPRi system, in which sgRNAs ought to be designed close to the initiation codon and target the non-template strand (Cao et al., 2017). We speculated the difference was because for PaeCascade, the whole cascade, containing multiple Cas7 proteins, was responsible to block transcription, while dCas9 functioned as the blocker alone in the type II CRISPRi system (Burstein et al., 2017; Ghavami and Pandi, 2021; Zheng et al., 2020). In this way, PaeCascade led to increased space steric hindrance, resulting in almost no position preference. As there was almost no limitation for the design of crRNA in the PaeCascade CRISPRi system, more spacers could be obtained than in type II CRISPRi. Therefore, it is easy to design effective crRNAs, which makes PaeCascade an outstanding tool for transcriptional inhibition in *S. oneidensis* MR-1.

### Transcription activation via PaeCascade-RpoD in *S. oneidensis* MR-1

**Figure 4:**
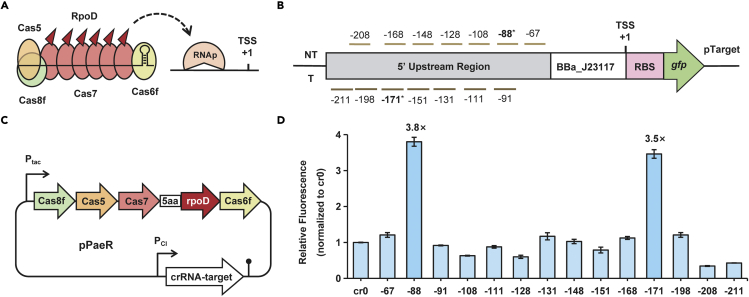
Transcription activation *via* PaeCascade-RpoD in *S. oneidensis* MR-1 (A) The schematic diagram and mechanism of PaeCascade-RpoD to recruit RNA polymerase (RNAp). RpoD (σ^70^) binds to transcriptional regulatory region and recruits the core RNAp (ɑ2ββ′ω) to enable gene activation. RpoD is fused to Cas7, responsible for binding protospacer. (B) Location sites of designed crRNAs targeting TSS upstream of *gfp* in the plasmid pTarget. (C) Plasmid map of PaeCascade-RpoD for transcriptional activation. The plasmid pPaeR is consisted of Cascade fused with RpoD proteins and a designed crRNA cassette controlled by P_tac_ and P_CI_ promoter, respectively. (D) The activation efficiency of type I-F PaeCascade-RpoD system targeting *gfp*. The values above the bar indicate the activation folds. Median GFP levels are normalized to the cr0 control strain. Values and error bars indicate mean ± SEM of three replicates. See also Figure S1.

We then aimed to employ PaeCascade as a transcriptional activation tool. It has been proven that RpoD (σ^70^) is able to bind to the transcriptional regulatory region and recruit the core RNA polymerase (ɑ2ββ′ω) to enable gene activation in bacteria (Dong et al., 2018). We thus selected RpoD (encoded by *rpoD* of *S. oneidensis* MR-1) as the activator. In addition, it has been reported that Cas7-activator fusion protein showed the highest transcription-activation dominance at targeted loci, compared to fusing with Cas5, Cas6f, or Cas8f (Luo et al., 2015). Therefore, the RpoD was C-terminally fused with Cas7 through five amino acid linkers (Figure 4A). To testify whether CRIPSR/PaeCascade-Cas7-RpoD system (hereafter PaeCascade-RpoD) could enable transcription activation in *S. oneidensis* MR-1, we adopted *gfp* in the plasmid pTarget as the reporter gene. Besides, to find the effective sites for upregulation, a well-designed sequence was applied with several PAM (5′-CC-3′) sites located in the upstream region of *gfp* as in the previous study (Dong et al., 2018). Then we designed a panel of crRNAs to target these sites from −211 bp to −67 bp upstream of the transcription start site (TSS) (Figure 4B). In our CRISPRa system, polycistronique cascade fused with RpoD was controlled by IPTG-induced promoter P_tac_, and crRNA was driven by constructive promoter P_CI_ in the plasmid pPaeR (Figure 4C). We noticed that significantly increased GFP expression occurred for sites at 88 bp (NT) and 171 bp (T) upstream of TSS, and the activation efficiency was 3.8-fold and 3.5-fold, respectively (Figure 4D). In the prior reports of the bacterial CRISPRa system, the efficiency of transcription activation depended on the distance of the crRNA targets from the TSS (Bikard et al., 2013). Similarly, this phenomenon of position dependence was also observed for PaeCascade-RpoD in *S. oneidensis* MR-1. Furthermore, we then attempted to promote the activation efficiency by adding a promoter P_tac_ in the forepart of Cas7-RpoD, allowing a higher expression level (Figure S1). However, the fluorescence intensity of GFP was not activated when targeting all these six sites (Figure S1). We thus employed the PaeCascade-RpoD without an additional promoter. Given the strong position dependence on upregulation, we recommended that the prioritized sites could be settled around 88 bp (NT) and 171 bp (T) upstream of TSS.

### Activation of cytochrome, electron shuttle, cell motility-related gene expression by PaeCascade-RpoD for enhancing extracellular electron transfer efficiency

**Figure 5:**
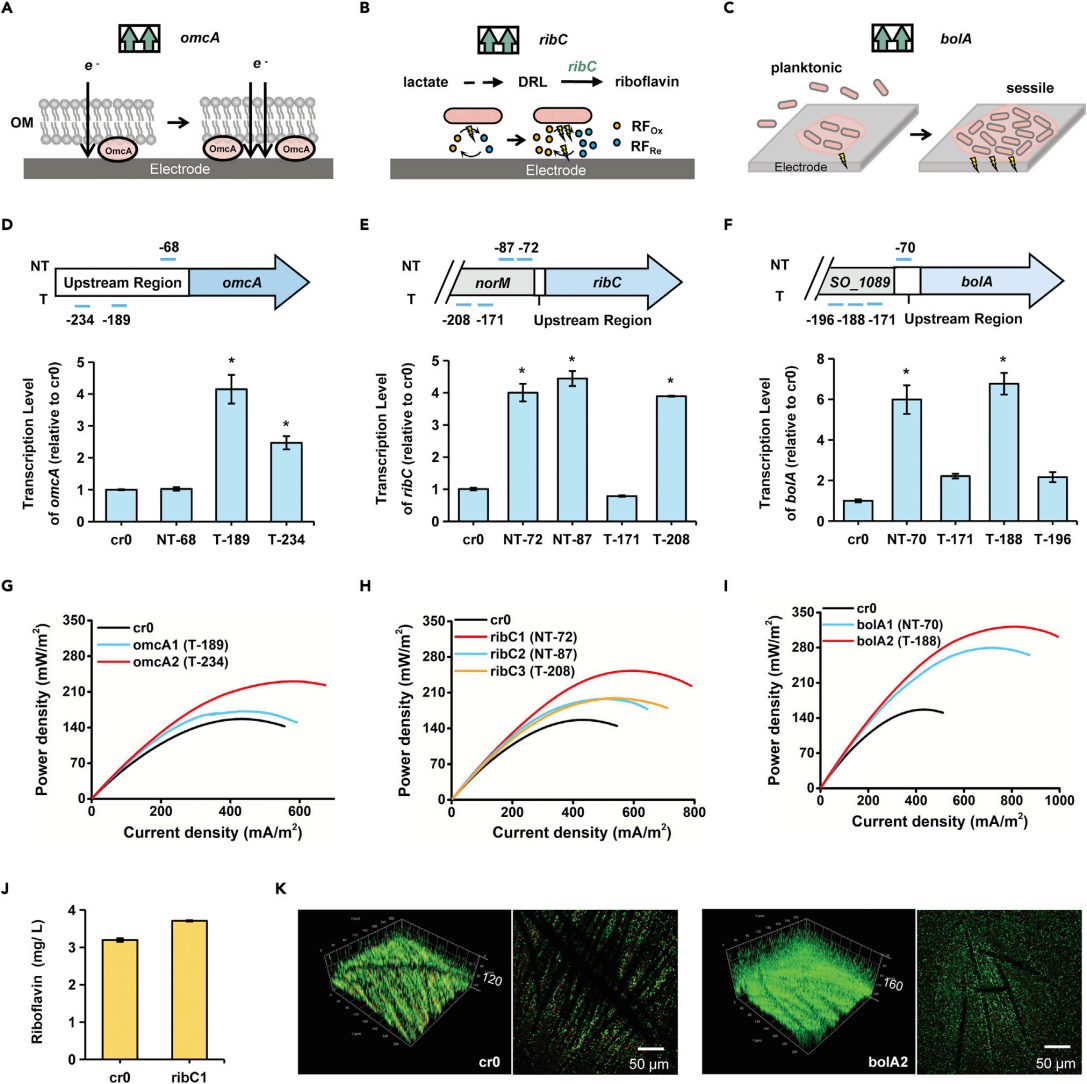
Activation of cytochrome, electron shuttle, cell motility-related gene expression by PaeCascade-RpoD for enhancing EET efficiency in *S. oneidensis* MR-1 (A–C) Schematic representation of (A) promoting OM c-Cyts by activating the expression of *omcA*, (B) modulating RF biosynthesis pathway to produce more RF by activating the expression of *ribC*, (C) transiting engineered strains from planktonic to the attachment stage by activating the expression of *bolA* to enhance the EET efficiency. (D–F) Targeting sites and corresponding transcriptional activation efficiency of (D) *omcA,* (E) *ribC,* and (F) *bolA* by qRT-PCR. *norM* and *SO_1089* are the adjacent upstream genes of *ribC* and *bolA*, respectively. Transcription levels are normalized to the control strain cr0. Values and error bars indicate mean ± SEM of three replicates. Asterisks mean the strains selected for the bio-electrochemical analysis in microbial fuel cells. (G–I) The power density output curves obtained by linear sweep voltammetry (LSV) with a scan rate of 0.1 mV/s of (G) *omcA,* (H) *ribC,* and (I) *bolA*-activated strains. cr0 is the control strain. See also Figure S2. (J) The production of riboflavin (RF) of the control strain cr0 and engineered strain ribC1. Values and error bars indicate mean ± SEM of three replicates. (K) Bio-image of strain cr0 and the activated strain bolA2 embedded on anode carbon cloth by Confocal Laser Scanning Microscope (CLSM). The number on the right of the biofilm means the bio-image thickness. All scale bars are 50 μm.

To demonstrate the application of PaeCascade-RpoD-mediated transcription activation for enhancing EET efficiency, the expression of three genes, *omcA, ribC,* and *bolA* were activated individually in *S. oneidensis* MR-1. OmcA, the outer membrane c-type cytochrome (OM c-Cyts), has been identified as a central player in transferring electrons from periplasm to electron acceptor (Figure 5A) (Meitl et al., 2009; Reardon et al., 2010). Riboflavin (RF) is the main electron shuttle of *Shewanella*, and RibC catalyzes the last step to synthesize RF from lactate (Figure 5B) (Yong et al., 2013). BolA is a motile/adhesive transcriptional switch, involved in the transition between the planktonic and the attachment stage of strains in the process of biofilm formation (Dressaire et al., 2015). Overexpression of *bolA* led to increased electroactive biomass, which is a crucial factor for electron transfer (Figure 5C) (Silva et al., 2020).

To activate the expression of *omcA, ribC* and *bolA*, we designed three to four crRNAs to target each gene. As shown in Figures 5D–5F, the qRT-PCR results demonstrated that the highest activation fold changes were 4.1 (T-189), 4.4 (NT-87), and 6.8 (T-188) of genes *omcA, ribC,* and *bolA*, respectively, compared with the no-targeting control strain cr0. Noteworthy, as the upstream regulatory regions of *ribC* and *bolA* are short, the designed crRNAs were in the coding sequence of their adjacent upstream genes. In spite of this, the transcription level of *ribC* and *bolA* were still successfully activated (Figures 5E and 5F). Then to identify the EET output of activated strains, we selected 2-3 strains with different upregulation fold changes for each gene and conducted bio-electrochemical analysis in microbial fuel cells (MFCs). The output voltages of the MFCs were presented in Figure S2. The highest voltages of *omcA, ribC,* and *bolA*-activated strains were 155.91 mV, 170.36 mV, and 221.51 mV, which were remarkably higher than that of the control strain cr0 (94.47 mV) (Figure S2). Besides, the linear sweep voltammetry (LSV) was performed during the plateau of voltage to obtain the power density. The maximum power densities of the strains omcA2, ribC1, bolA2 were up to 230.46 mW/m^2^ and 252.96 mW/m^2^ and 321.67 mW/m^2^. The EET efficiencies showed ∼1.5, ∼1.6, and ∼2.1 times enhancement than the strain cr0 (156.67 mW/m^2^), respectively (Figures 5G–5I). Furthermore, corresponding phenotype changes in the activated strains were also observed. The RF production of strain ribC1 was 3.709 mg/L, 0.51 mg/L higher than that of strain cr0. The results indicated slight improvement in the RF production, leading to the enhanced ability of electron transfer (Figures 5H and 5J). We next investigated whether the biofilm of strain bolA2 was increased by utilizing confocal laser scanning microscope (CLSM) on the MFC carbon cloth. As shown in Figure 5K, the biofilm of strain bolA2 was obviously denser and thicker than that of strain cr0, causing the drastically improved EET efficiency. Collectively, endogenous genes were successfully upregulated by the PaeCascade-RpoD-mediated CRISPRa system, resulting in positive effects on the electron generation capacity of *S. oneidensis* MR-1 in MFCs.

### Inhibition of biofilm, cell morphology-related gene expression by PaeCascade-RpoD for enhancing extracellular electron transfer efficiency

**Figure 6:**
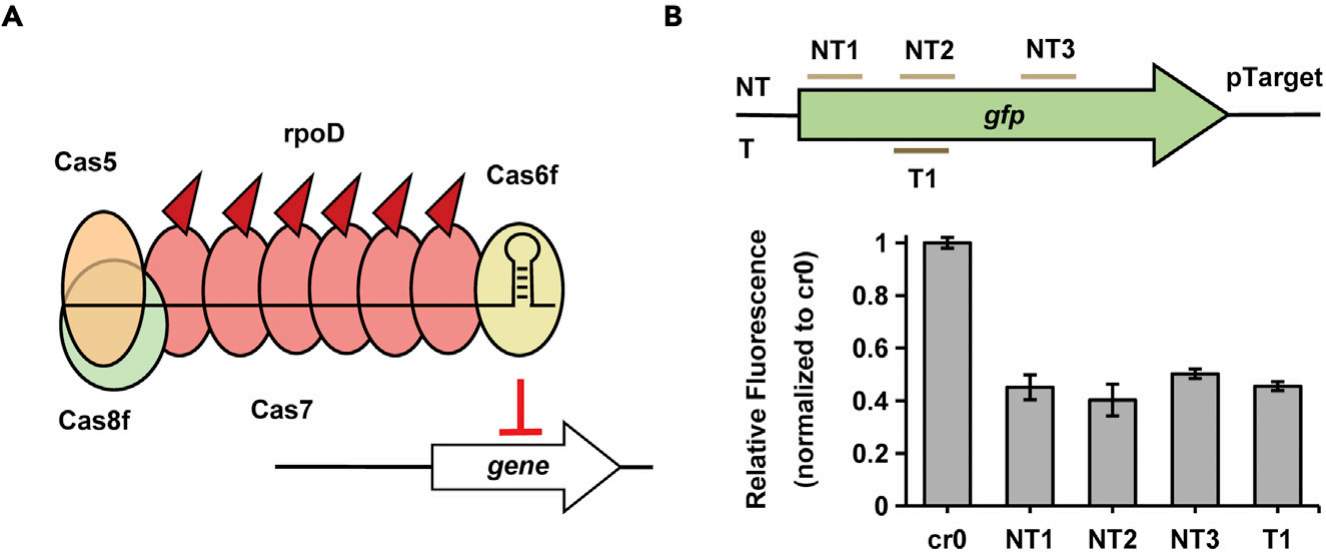
PaeCascade-RpoD system also successfully enabled transcriptional inhibition in *S. oneidensis* MR-1 (A) The schematic diagram of gene repression by PaeCascade-RpoD. (B) Transcriptional inhibition was enabled by PaeCascade-RpoD targeting *gfp*. Targeting sites of designed crRNAs of *gfp* in the plasmid pTarget and the inhibition efficiency of type I-F PaeCascade-RpoD system. Median GFP levels are normalized to the control strain cr0. Values and error bars indicate mean ± SEM of three replicates.

Although activators recruit the RNAp complex, transcription inhibition can still be achieved by CRISPR/dCas9-activator system when targeting the open reading frame (ORF) instead of upstream of promoter (Bhokisham et al., 2020). We then assessed whether our PaeCascade-RpoD system could also be employed as the tool of CRISPRi, and four crRNAs were designed to target the ORF of *gfp* (Figure 6A). As shown in Figure 6B, the expression of *gfp* was repressed when targeting all these four loci, from 2-fold to 2.5-fold, indicating that PaeCascade-RpoD remained the interference capability and there was also almost no position dependence as discovered for PaeCascade-mediated gene repression (Figure 3B). In consequence, it is flexible to employ PaeCascade-RpoD as both transcription activation and inhibition tool, and thus the following application utilized PaeCascade-RpoD for gene repression in *S. oneidensis* MR-1.

**Figure 7:**
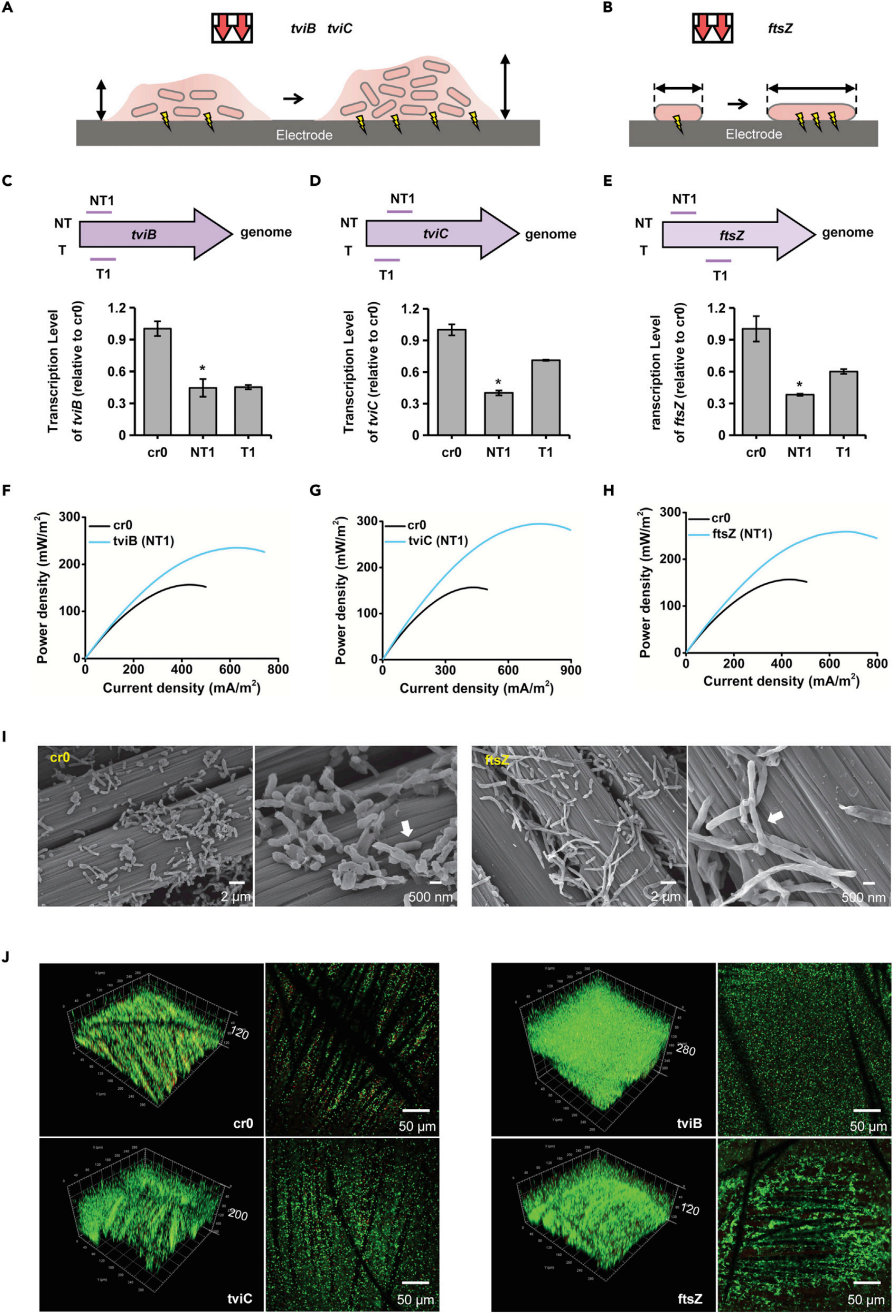
Inhibition of biofilm, cell morphology-related gene expression by PaeCascade-RpoD for enhancing EET efficiency in *S. oneidensis* MR-1 (A and B) Schematic representation of (A) engineering the thickness and morphology of biofilm by repressing the expression of *tviB* or *tviC*, (B) engineering the cell morphology by repressing the expression of *ftsZ* to enhance the EET efficiency. (C–E) Targeting sites and corresponding transcriptional inhibition efficiency of (C) *tviB,* (D) *tviC*, and (E) *ftsZ* by qRT-PCR. Transcription levels are normalized to the control strain cr0. Values and error bars indicate mean ± SEM of three replicates. Asterisks mean the strains selected for the bio-electrochemical analysis in microbial fuel cells. (F–H) The power density output curves obtained by linear sweep voltammetry (LSV) with a scan rate of 0.1 mV/s of (F) *tviB,* (G) *tviC*, and (H) *ftsZ*-repressed strains. cr0 is the control strain. See also Figure S3. (I) Scanning electron microscope image of the strain cr0 and repressed strain ftsZ (NT1) on anode carbon cloth. Scale bars are 2 μm for low magnification views, and 500 nm for high magnification views. (J) Bio-image of strain cr0 and the repressed strains tviB (NT1), tviC (NT1), ftsZ (NT1) embedded on anode carbon cloth by CLSM. The number on the right of the biofilm means the bio-image thickness. All scale bars are 50 μm.

To demonstrate the application of PaeCascade-RpoD-mediated CRISPRi for enhancing EET efficiency, the expression of three genes, *tviB*, *tviC,* and *ftsZ* were repressed separately in *S. oneidensis* MR-1. Biofilm-related genes *tviB* and *tviC* were selected from the cell surface polysaccharide biosynthesis gene cluster (Kouzuma et al., 2010). It has been reported that base editing-mediated deactivation of these two genes increased the thickness and density of biofilm formed by EAMs on electrode surfaces (Chen et al., 2022), which has a positive effect on both cytochrome-mediated direct EET and shuttle-related indirect EET pathways (Edel et al., 2019). We thus conjectured that interference of *tviB* and *tviC* expression might cause thicker and/or denser biofilm, contributing to the improvement of the EET efficiency (Figure 7A). FtsZ participates in the process of cell division through self-polymerization, responsible for the formation of a Z ring in the middle of the cell (Bi et al., 1991). Accordingly, we speculated that the inhibition of *ftsZ* expression may lead to interfering with the formation of the Z ring, resulting in the longer cell morphology of *S. oneidensis* MR-1 and directly increasing the contact area between EAMs and electron acceptor (Figure 7B).

To repress the expression of *tviB*, *tviC,* and *ftsZ*, we designed two crRNAs (NT1 and T1) to target the ORF of each gene. As shown in Figures 7C–7E, the qRT-PCR results demonstrated that the higher transcriptional inhibition efficiency of *tviB*, *tviC,* and *ftsZ*-repressed strains were 1.75-, 2.49-, and 2.62-fold, between the two targets of each gene. And next these three strains were incubated in anodic chambers of MFCs for bio-electrochemical analysis. The output voltages of *tviB*, *tviC,* and *ftsZ*-repressed strains were 156.49 mV, 187.04 mV, and 171.54 mV, indicating a large increase over the control strain cr0 (94.47 mV) in the MFCs (Figure S3). The LSV was conducted during the plateau of voltage. The maximum power density of the strains tviB (NT1), tviC (NT1), and ftsZ (NT1) reached 234.97 mW/m^2^ and 294.63 mW/m^2^ and 259.10 mW/m^2^, respectively, showing ∼1.5, ∼1.9 and ∼1.7 times higher than that of the strain cr0 (156.67 mW/m^2^) (Figures 7F–7H). The results indicated that the PaeCascade-RpoD-mediated CRISPRi system significantly improved the EET efficiency in *S. oneidensis* MR-1 by repressing these three genes’ expression. Then scanning electron microscope was conducted to investigate the effect on cell morphology of repressing the expression of *ftsZ*. The strain ftsZ (NT1) showed obviously longer morphology than strain cr0 on carbon cloth in MFCs (Figure 7I). We also performed CLSM on the MFC carbon cloth electrodes of the cr0 and the repressed strains to detect the phenotype of the biofilm. The biofilm of strains tviB (NT1) and tviC (NT1) showed a remarkably thicker and more compact structure than cr0 strain (Figure 7J). In addition, more biomass was adhered on the carbon fiber of strains tviC (NT1) and ftsZ (NT1) than strain cr0, suggesting high-level adhesiveness of engineered strains to anodes (Figure 7J). Based on the above bio-imaging results, repression of cell division- and polysaccharide synthesis-related genes led to the expected changes in phenotypes, including cell morphology, biofilm thickness/density, and cell-anode adhesiveness, thereby enhancing the electron transfer capacity of the engineered strains in the MFCs. Therefore, the PaeCascade-RpoD-mediated CRISPRi system demonstrated the outstanding ability of transcription inhibition of native genes, and contributed to improved EET efficiency in *S. oneidensis* MR-1.

### Simultaneous activation and inhibition by PaeCascade-RpoD in *S. oneidensis* MR-1

**Figure 8:**
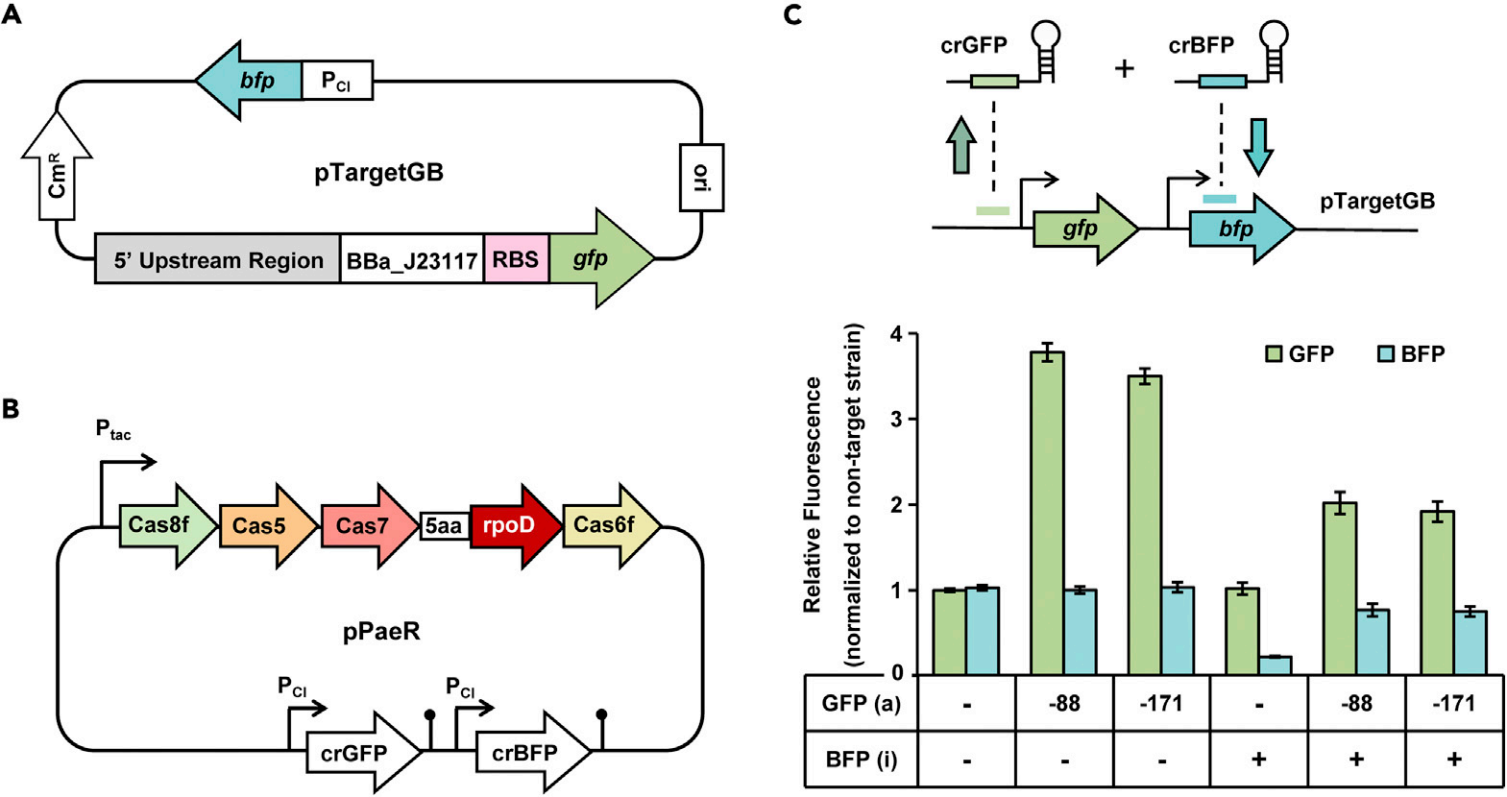
Simultaneous activation and inhibition by PaeCascade-RpoD in *S. oneidensis* MR-1 (A) Plasmid map of pTargetGB for simultaneous regulation. (B) Plasmid map of pPaeR containing two crRNA expression cassettes for activating GFP and inhibiting BFP. (C) Schematic diagram and simultaneous regulation efficiency of PaeCascade-RpoD. GFP (a) indicates GFP activation, and BFP (i) indicates BFP inhibition. “+” stands for performing corresponding regulation, and “-” stands for without corresponding regulation. “-88” or “-171” means the targeting position for activating GFP. Median fluorescence is normalized to the non-target strain. Values and error bars indicate mean ± SEM of three replicates.

On the basis of PaeCascade-RpoD enabling sole activation or inhibition, the possibility of simultaneous modulation was further testified. We attempted to activate GFP and repress BFP at the same time, and *gfp* and *bfp* were constructed in one plasmid pTargetGB (Figure 8A). Then two crRNAs targeting *gfp* and *bfp* were assembled into another plasmid pPaeR (Figure 8B). As shown in Figure 8C, the fluorescence of *gfp* was increased by ∼2.0-fold or ∼1.9-fold targeting 88 bp (NT) or 171 (T) upstream of TSS, and the expression of *bfp* was decreased by ∼1.3-fold targeting ORF. The results indicated that PaeCascade-RpoD was able to achieve gene activation and inhibition at the same time. The fold changes in simultaneous regulation were lower than that of separate regulation (∼3.8-fold for sole activation and ∼4.5-fold for sole repression) (Figure 8C). We speculated the possible reason was that two crRNAs competed for a fixed pool of Cas proteins, leading to the insufficient supply of Cascade for activating GFP and inhibiting BFP. In sum, PaeCascade-RpoD has the potential to perform orchestra regulation easily by incorporating multiple crRNAs, providing a powerful dual-modulation tool in *S. oneidensis*.

## Discussion

In this work, the transcriptional activation was achieved by CRISPRa in *S. oneidensis*. To our knowledge, the previous approaches to increase gene expression level in *S. oneidensis* MR-1 is overexpression based on plasmids and genomic knock-in (Fan et al., 2021a, 2021b; Yi and Ng, 2021). These methods are labor-intensive and time-consuming, owing to the difficulty of plasmid construction, inevitable steps of codons optimization and appropriate promoter selection to achieve specially adapted strength for the EET improvement (Cao et al., 2019). For the PaeCascade-RpoD-mediated CRISPRa system implemented in this study, only 32-bp crRNA needs to be designed and assembled into plasmid through the Golden Gate strategy to enhance the transcription level of target genes. Thus, PaeCascade-RpoD makes it easier to obtain regulated strains with high EET efficiency rapidly.

Furthermore, this novel type I-F CRISPR/PaeCascade-RpoD-mediated activation and inhibition regulation (CRISPR-PAIR) platform not only enables transcriptional activation readily, but also achieves transcriptional inhibition and even simultaneous modulation through a single system in *S. oneidensis* MR-1. Compared to the previous CRISPRi tools (Cao et al., 2017; Li et al., 2020) and PaeCascade system without RpoD, CRISPR-PAIR platform facilitates multi-mode and multi-gene regulation more conveniently. Besides, genetic up-regulation and down-regulation tools can be used as Amplicon and NOT-gate in genetic circuits (Santos-Moreno and Schaerli, 2020; Wall et al., 2004). In this way, combining our CRISPR-PAIR platform with biosensors could shed light on the dynamical control of the substance and energy metabolism automatically.

For the practical applications of MFCs, the use of antibiotics and inducers is detrimental. To circumvent this problem, it is a good choice to replace the inducible promoter with a constitutive promoter and insert our system into the genome of *S. oneidensis* MR-1. Accordingly, gene activation and inhibition are able to be implemented without the addition of antibiotics and inducible agents. Meanwhile, in the context of EET fundamental research, the CRISPR-PAIR platform is an ideal tool to identify the relationship between genes and electron transfer, offering the potential of large-scale modulation to inspire mechanistic studies of EET in *S. oneidensis*.

### Limitations of the study

Type I-F Cascade (*cas8f*, *cas5*, *cas7,* and *cas6f*) gene sequences used in plasmids pPaeCascade and pPaeR were directly obtained from the plasmid pCsy_complex. That is to say, the sequences were not codon-optimized, which might result in the suboptimal effect of gene activation and inhibition via CRISPR-PAIR platform.

## STAR★Methods

### Key resources table

**Table:** 

REAGENT or RESOURCE	SOURCE	IDENTIFIER
**Bacterial and virus strains**		

*E. coli* DH5α	Lab stock	N/A
*E. coli* Trans1-T1	Lab stock	N/A
*E. coli* WM3064	Lab stock	N/A
*S. oneidensis* MR-1	Lab stock	N/A
*S. oneidensis* GZ	Cheng et al., 2020	N/A

**Chemicals, peptides, and recombinant proteins**		

60% Sodium lactate solution	ACMEC Biochemical	S58050
2,6-Diaminopimelic acid	Yuanye Biology	S30747
Kanamycin Sulfate	Solarbio	Cat#K8020
Chloromycetin	Solarbio	Cat#C8050
Potassium ferricyanide(Ⅲ)	J&K Scientific	Cat#911115

**Critical commercial assays**		

RNAprep pure Cell/Bacteria Kit	TIANGEN	Cat#DP430
RevertedAid First Strand cDNA Synthesis Kit	Thermo Fisher Scientific	Cat#K1622
SYBR Green super mix	AB clonal	Cat#RM21203
LIVE/DEAD^TM^ Baclight^TM^ bacterial Viability Kit	Invitrogen (Thermo Fisher)	Cat#L7012

**Oligonucleotides**		

All oligonucleotides used as guide sequences or for qRT-PCR and plasmid construction are listed in supplemental materials.	N/A	N/A

**Recombinant DNA**		

All plasmids used in this study are listed in supplemental materials.	N/A	N/A

### Resource availability

#### Lead contact

Further information and requests for resources and reagents should be directed to and will be fulfilled by the lead contact, Yingxiu Cao (caoyingxiu@tju.edu.cn).

#### Materials availability

All requests for strains and plasmids constructed in this study should be directed to lead contact, Yingxiu Cao (caoyingxiu@tju.edu.cn).

### Experimental model and subject details

*E. coli* DH5α and Trans-T1 were employed for cloning and cultivated aerobically at 37°C in Luria-Bertani (LB) broth. *E. coli* WM3064 was used to carry out Golden Gate Assembly and transform plasmids into *S. oneidensis* MR-1*. E. coli* WM3064 was cultivated in LB broth with 0.3 mM 2,6-Diaminopimelic acid (DAP). Kanamycin (Km, 50 μg/mL) or chloramphenicol (Cm, 34 μg/mL) was added to LB broth as required. *S. oneidensis* MR-1 and *S. oneidensis* GZ strains were cultured aerobically at 30 °C in LB broth. Km (50 μg/mL), Cm (34 μg/mL), and isopropyl-β-D-thiogalactopyranoside (IPTG, 0.8 mM for P_tac_) were added in the medium as required.

### Method details

#### Plasmid construction and crRNAs design

The plasmids used in this study are listed in Table S1.

pPaeCascade was constructed by Genewiz with *cas8f (csy1), cas5 (csy2), cas7 (csy3), cas6f (csy4)* sequence from pCsy_complex, gift from Prof. Puping Liang (Chowdhury et al., 2017) and crRNA cassette integrated to pCYR011 (Chen et al., 2022). To facilitate rapid construction of crRNA, we introduced Golden Gate assembly strategy (Engler et al., 2008; Fang et al., 2021), so the crRNA cassette contained repeat sequences and spacer sequence with BsaI recognition sites synthesized by Genewiz (China). For Golden Gate assembly, a 17-bp base sequence is between the repeats of PaeCascade handle: GAAA-AgagaccAAAggtctcG-GTTC (lowercase letters represent the BsaI recognition site) in the crRNA expression cassette. The sticky ends of -CTTT and GTTC- were generated with the cleavage by BsaI. Besides, the designed spacer primers were annealed to form a 32-bp DNA fragment with a 4-bp sticky end, which was just complementary to -CTTT and GTTC- produced by BsaI in the crRNA expression cassette in the plasmid. Then 32-bp spacer sequence was connected to the plasmid by DNA ligation in one pot (Fang et al., 2021). pSpuCascade was constructed with *cas7fv, cas5fv, cas6fv* sequences and corresponding rRNA cassette synthesized by Genewiz (China) integrated to pCYR011. pGsuCascade was constructed with *cas8u2, cas7, cas5* sequences and corresponding crRNA cassette synthesized by Genewiz (China) integrated to pCYR011. To generate pPaeR, the linker sequence (-ggtggtggtggttct-) and rpoD sequence were connected to C-terminus of Cas7 derived from pPaeCascade. pPaeR-cr0 were constructed by removing the spacer sequence from pPaeR utilizing 2×Seamless Cloning Mix (Biomed, China). pTarget was constructed by Genewiz with J1 upstream region (Dong et al., 2018), promoter J23117 and *gfp* sequence integrated to pEWTEST1 (Meitl et al., 2009). Sequences of the primers for plasmid construction, genome amplification and sequencing are listed in Table S2. Sequences of PaeR and crRNA cassette used in this study are listed in Table S3.

#### Fluorescence assay in *S. oneidensis* MR-1

GFP were employed to characterize expression intensity. The promoter P_tac_ was induced by 0.8 mM IPTG. When *gfp* was in the plasmid pTarget, chloramphenicol (Cm, 34 μg/mL) was needed to add to LB broth. For fluorescence intensity assay, every strain inoculated from a fresh colony on an LB agar plate was cultured in LB medium (2 ml) with corresponding antibiotics for 12 h as a seed culture. Then 50 μl of seed culture was added to 5 ml LB medium with the proper antibiotic and the corresponding concentration of inducer in test tubes. After 24-h incubation with constant shaking (200 rpm) at 30°C, 50 μl suspensions from each test tube were centrifuged at 5000 rpm for 8 min to remove the supernatant. Then we resuspended the culture with 500 μl of phosphate-buffered saline (PBS) and transferred 200 μl of suspension to a 96-well polystyrene plate (black plat) (Greiner bio-one μclear, Germany). Cell optical density and fluorescence intensity were detected by a Tecan Infinite200 M Plex microplate reader. Optical density was measured at 600 nm. The excitation/emission wavelength was set at 485 nm/520 nm for GFP, and 399 nm/456 nm for BFP. Assays were conducted in triplicate, and *S. oneidensis* MR-1 strains with pPaeR-cr0 were used as control. Relative fluorescence was calculated as the following equation.

RelativeFluorescence=Fluorescence(a/i)OD600(a/i)Fluorescence(control)OD600(control)

#### Quantitative real-time reverse transcription polymerase chain reaction (qRT-PCR)

Total RNA was extracted from the strains in mid-log phase through a RNAprep pure Cell/Bacteria Kit (TIANGEN, China) on the basis of the manufacturer’s instructions. Besides, cDNA was obtained *via* the RevertedAid First Strand cDNA Synthesis Kit (Thermo Fisher Scientific, the USA). Quantitative analysis of gene expression was conducted by SYBR Green super mix (AB clonal, the USA). The *gyrB* gene of *S. oneidensis* MR-1 was utilized for normalization. Samples were tested in triplicate with the listed primers (Table S3). PCR conditions consisted of denaturing at 95°C for 1 min, and 40 cycles of denaturing at 95°C for 15 s followed by annealing and extension at 60°C for 30 s. Relative gene expression was calculated using the method 2^−ΔΔC^_T_, normalized with the reference gene *gyrB*.

#### Bio-electrochemical characterization

*omcA, ribC, bolA*-activated strains, and *tviB, tviC, ftsZ*-inhibited strains and cr0 strain were inoculated from a fresh single colony on an LB agar plate with Km (50 μg/mL) into 2 ml LB medium with Km (50 μg/mL) as a seed culture. 1 mL seed was added into 100 mL fresh LB broth with Km (50 μg/mL) and IPTG(0.8 mM) at 30°C with shaking (200 rpm). After about 10-h culture, we adjusted the concentrations of cell suspensions to the same level (OD_600_ = 0.5). MFCs were incubated in a 30°C incubator conducted in triplicate, and *S. oneidensis* MR-1 strains with pPaeR-cr0 were used as control. Dual-chamber MFCs with 140 mL as working volume separated through the Fanion 117 membrane (DuPont Inc., the USA) were employed in this study. Carbon cloth was utilized as the electrodes for cathode (2.5 cm × 3 cm) and anode (1 × 1 cm, the geometric area is 1 cm^2^). The anolyte was composed of M9 buffer (NaCl, 0.5 g/L; NH_4_Cl, 1 g/L; KH_2_PO_4_, 3 g/L; Na_2_HPO_4_, 6 g/L; CaCl_2_, 0.1 mM MgSO_4_, 1 mM), supplemented with 20 mM lactate, 5% (v/v) LB broth, 1 mg/L Kan and 1 mM IPTG. The catholyte constituted 50 mM KH_2_PO_4_, 50 mM K_3_[Fe (CN)_6_], and 50 mM K_2_HPO_4_ solution. To measure the voltage generation, we connected a 2k external resistor with the external circuits of MFCs, and the output voltages were recorded. Linear sweep voltammetry (LSV) analysis with a scan rate (0.1 mV/s) was performed on a two-electrode mode. The anode was used as the working electrode with the cathode as the reference. The electrode got the polarization curves to estimate the maximum power density. Power density (P) was calculated as P = V (output voltage) × I (current density). Both I and P were normalized to the projected area of the anode surface.

#### Quantification of extracellular flavin

*ribC*-activated strain and cr0 strain were inoculated from a fresh single colony on an LB agar plate with Km (50 μg/mL) into 2 mL LB medium with Km (50 μg/mL) as a seed culture. 0.5 mL seed was added into 50 mL fresh LB broth with Km (50 μg/mL) and IPTG (0.8 mM) at 30°C with shaking (200 rpm). After 12-h fermentation, 1 mL suspensions were centrifuged at 10000 rpm for 10 min to obtain the supernatant. Then the supernatant was filtered through membrane (0.22 mm) and riboflavin (RF) in the supernatant was determined by high-performance liquid chromatography (HPLC, Shimadzu Corporation).

#### Bio-imaging

For strain cr0 and ftsZ(i), after LSV synthesis was conducted, we cut the carbon cloth, soaked it in 2.5% glutaraldehyde, and put it in the refrigerator at 4°C overnight. Then after pouring out the glutaraldehyde, the carbon cloth was soaked in the PBS for three times (8 min/time). Next, the carbon cloth was soaked in 30%, 50%, 70%, 80%, and 90% ethanol solutions (8 min/time), respectively. Finally, vacuum freeze-drying was conducted for 10 h, and the bacteria on the carbon cloth were imaged by scanning electron microscope.

For strain cr0, bolA, tviB, tviC, ftsZ, anode carbon cloth was dyed by LIVE/DEAD^TM^ Baclight^TM^ bacterial Viability Kit. Then the carbon cloth was imaged to observe biofilm thickness and morphology by Confocal Laser Scanning Microscope (CLSM).

### Quantification and statistical analysis

Results are reported as values with error bars, which indicate mean ± SEM of technical triplicates in the figure legends. SEM indicates the standard error of the mean. The figures of power density output are drawn by Origin software. The CLSM bio-imaging was exported by ZEN software.

## Data Availability

• All data reported in this paper will be shared by the lead contact upon request. • This paper does not report original code. • Any additional information required to reanalyze the data reported in this paper is available from the lead contact upon request.
