# Supplementary material for: Type I-F CRISPR-PAIR platform for multi-mode regulation to boost extracellular electron transfer in Shewanella oneidensis
Source: iScience. 2022 May 30;25(6):104491. doi: 10.1016/j.isci.2022.104491 (PMC9194131; doi:10.1016/j.isci.2022.104491)
Supplement: Document S1. Figures S1–S3 and Table S1 — –S4 [file mmc1.pdf]

iScience, Volume *25*

Supplemental information

Type I-F CRISPR-PAIR platform for multi-mode regulation to boost extracellular electron transfer in *Shewanella oneidensis*

Yaru Chen, Meijie Cheng, Hao Song, and Yingxiu Cao

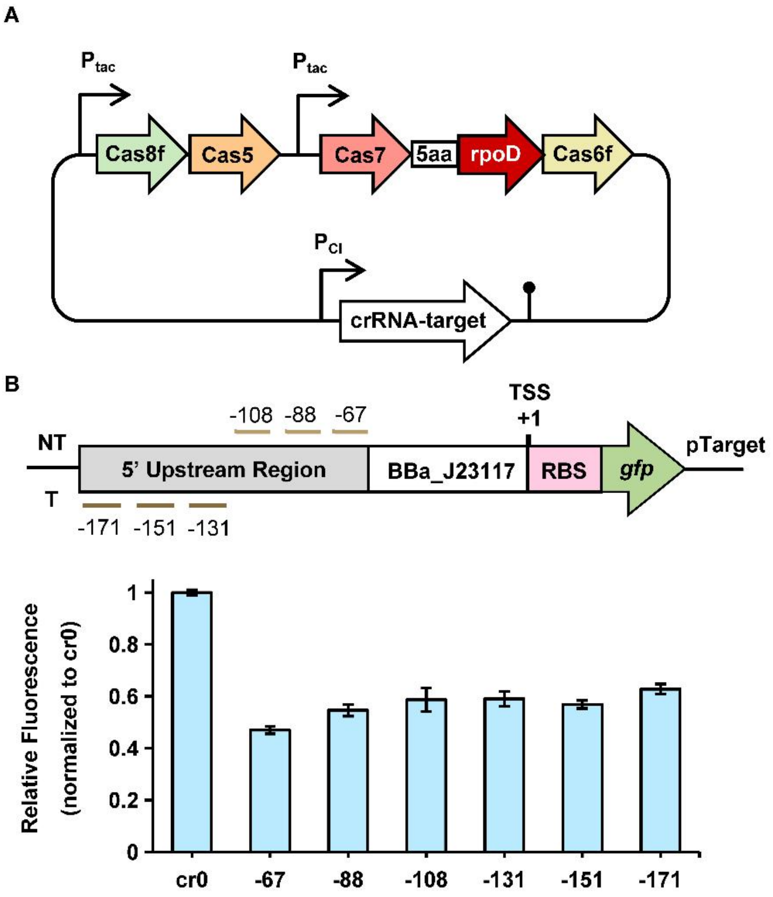

Figure S1. Adding a promoter P_{tac} in the forepart of Cas7-RpoD caused failed activation in *S. oneidensis* MR-1, related to Figure 4.

(A) Plasmid map of the PaeCascade-RpoD system for transcriptional activation with an added promoter P_{tac} in the forepart of Cas7-RpoD.

(B) Location sites of designed crRNAs targeting TSS upstream of *gfp* in the plasmid pTarget and the activation efficiency. Median GFP levels are normalized to the cr0 control strain. Values and error bars indicate mean +/- standard error of mean (SEM) of three replicates.

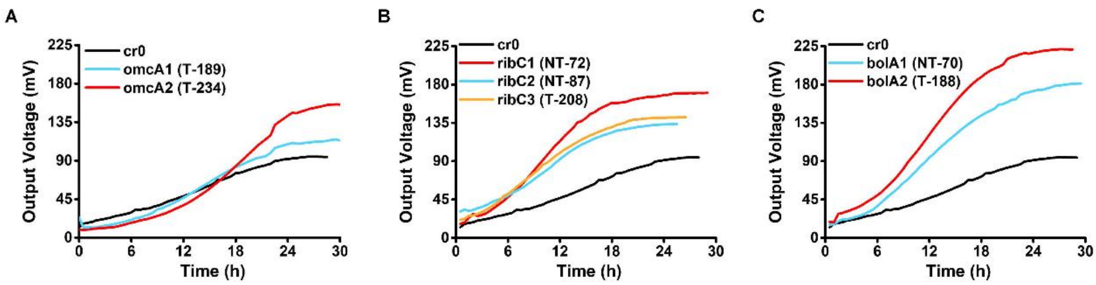

Figure S2. The output voltages of (A) *omcA*, (B) *ribC* and (C) *bolA*-activated strains in the MFCs, related to Figure 5. cr0 is the control strain.

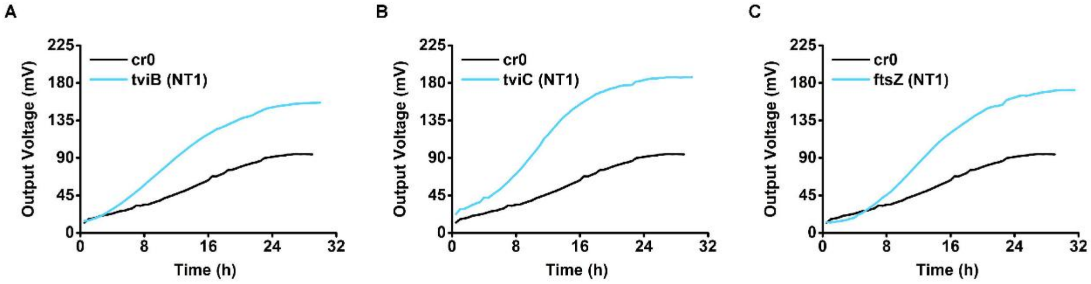

Figure S3. The output voltages of (A) *tviB*, (B) *tviC* and (C) *ftsZ*-inhibited strains in the MFCs, related to Figure 7. cr0 is the control strain.

Table S1. Plasmids used in this study, related to STAR Methods.

Plasmid [b)]	Description[a)]	Reference or source
pCsy_complex	ori, bom, rop, Amp^R, pT7, *cas8f* (*csy1), cas5 (csy2), cas7 (csy3), cas6f (csy4)*	(Chowdhury et al., 2017)
pCYR011	pTac, reppBBR1, Kan^R, oriT, *gfp*	(Chen et al., 2022)
pPaeCascade	pCYR011 derivative, *cas8f, cas5, cas7, cas6f*, crRNA	This study
pSpuCascade	pCYR011 derivative, *cas7fv, cas5fv, cas6fv*, crRNA	This study
pGsuCascade	pCYR011 derivative, *cas8u2, cas7, cas5*, crRNA	This study
pPaeR	pPaeCascade derivative, *cas7-ggtggtggtggttct-rpoD*	This study
pPaeR-cr0	pPaeCascade-RpoD derivative, *cas7-ggtggtggtggttct-rpoD*, crRNA cassette removal of spacer sequence	This study
pEWTEST1	pBAD, repCM62, Cm^R, oriT	(Chen et al., 2022)
pTarget	pEWTEST1 derivative, J1 upstream region, pJ23117, *gfp*	This study
pTargetGB	pTarget derivative, P_{CI}, *bfp*	This study

[a)] Amp^R, Km^R and Cm^R represent resistance to ampicillin, kanamycin and chloramphenicol, respectively;

[b)] crRNA expression plasmids with changed guide sequences are not listed here. Lists of guide sequences used in this study are shown in Table S2.

Table S2. Primers of guide sequences used in this study, related to STAR Methods.

Primer	Sequence
P.ae-cr1-F	GAAAGTATGTTGCATCACCTTCACCCTCTCCACTGA
P.ae-cr1-R	GAACTCAGTGGAGAGGGTGAAGGTGATGCAACATAC
P.ae-cr2-F	GAAATGTACGTAACCTTCGGGCATGGCACTCTTGAA
P.ae-cr2-R	GAACTTCAAGAGTGCCATGCCCGAAGGTTACGTACA
P.ae-cr3-F	GAAACTTGTTAATAGAATCGAGTTAAAAGGTATTGA
P.ae-cr3-R	GAACTCAATACCTTTTAACTCGATTCTATTAACAAG
S.pu-cr1-F	GAAAGTATGTTGCATCACCTTCACCCTCTCCACTGA
S.pu-cr1-R	GAACTCAGTGGAGAGGGTGAAGGTGATGCAACATAC
S.pu-cr2-F	GAAATGTACGTAACCTTCGGGCATGGCACTCTTGAA
S.pu-cr2-R	GAACTTCAAGAGTGCCATGCCCGAAGGTTACGTACA
S.pu-cr3-F	GAAACTTGTTAATAGAATCGAGTTAAAAGGTATTGA
S.pu-cr3-R	GAACTCAATACCTTTTAACTCGATTCTATTAACAAG
G.su-cr1-F	GAAATCCGTATGTTGCATCACCTTCACCCTCTCCACTGAC
G.su-cr1-R	GAACGTCAGTGGAGAGGGTGAAGGTGATGCAACATACGGA
G.su-cr2-F	GAAACCTGTACGTAACCTTCGGGCATGGCACTCTTGAAAA
G.su-cr2-R	GAACTTTTCAAGAGTGCCATGCCCGAAGGTTACGTACAGG
G.su-cr3-F	GAAAAAGGTGATACCCTTGTTAATAGAATCGAGTTAAAAG
G.su-cr3-R	GAACCTTTTAACTCGATTCTATTAACAAGGGTATCACCTT
P1-F	GAAACCAGGCTTTACACTTTATGCTTCCGGCTCGTA
P1-R	GAACTACGAGCCGGAAGCATAAAGTGTAAAGCCTGG
P2-F	GAAAACACAACATACGAGCCGGAAGCATAAAGTGTA
P2-R	GAACTACACTTTATGCTTCCGGCTCGTATGTTGTGT
NT1-F	GAAAACTGACAGAAAATTTGTGCCCATTAACATCAC
NT1-R	GAACGTGATGTTAATGGGCACAAATTTTCTGTCAGT
NT2-F	GAAAGTATGTTGCATCACCTTCACCCTCTCCACTGA
NT2-R	GAACTCAGTGGAGAGGGTGAAGGTGATGCAACATAC
NT3-F	GAAAGAAATTCAAGAGTGCCATGCCCGAAGGTTACGTACA
NT3-R	GAACTTCAAGAGTGCCATGCCCGAAGGTTACGTACA
NT4-F	GAAATTTTAACTCGATTCTATTAACAAGGGTATCAC
NT4-R	GAACGTGATACCCTTGTTAATAGAATCGAGTTAAAA
NT5-F	GAAAATCTTCAATGTTGTGTCTAATTTTGAAGTTAA
NT5-R	GAACTTAACTTCAAAATTAGACACAACATTGAAGAT
NT6-F	GAAAAATCGGAGTATTTTGTTGATAATGGTCTGCTA
NT6-R	GAACTAGCAGACCATTATCAACAAAATACTCCGATT
T1-F	GAAAAATTCTTGTTGAATTAGATGGTGATGTTAATG
T1-R	GAACCATTAACATCACCATCTAATTCAACAAGAATT
T2-F	GAAATTAAATTTATTTGCACTACTGGAAAACTACCT
T2-R	GAACAGGTAGTTTTCCAGTAGTGCAAATAAATTTAA
T3-F	GAAAGTGGCCAACACTTGTCACTACTTTCGGTTATG
T3-R	GAACCATAACCGAAAGTAGTGACAAGTGTTGGCCAC

T4-F	GAAAAGATCACATGAAACAGCATGACTTTTTCAAGA
T4-R	GAACTCTTGAAAAAGTCATGCTGTTTCATGTGATCT
T5-F	GAAAATGCCCGAAGGTTACGTACAGGAAAGAACTAT
T5-R	GAACATAGTTCTTTCCTGTACGTAACCTTCGGGCAT
T6-F	GAAACTTGTTAATAGAATCGAGTTAAAAGGTATTGA
T6-R	GAACTCAATACCTTTTAACTCGATTCTATTAACAAG
T7-F	GAAATTGTTAATAGAATCGAGTTAAAAGGTATTGAT
T7-R	GAACATCAATACCTTTTAACTCGATTCTATTAACAA
-67-F	GAAACAAAGGACGCCTTTGGTAACCGCAGGACACCG
-67-R	GAACCGGTGTCCTGCGGTTACCAAAGGCGTCCTTTG
-88-F	GAAAGCAGGACACCGCAGGATACCTGAGGTCGCCCG
-88-R	GAACCGGGCGACCTCAGGTATCCTGCGGTGTCCTGC
-91-F	GAAATGCGGTTACCAAAGGCGTCCTTTGGGTTCCAC
-91-R	GAACGTGGAACCCAAAGGACGCCTTTGGTAACCGCA
-108-F	GAAATGAGGTCGCCCGTGGTGGCCCATGGTCACCAT
-108-R	GAACATGGTGACCATGGGCCACCACGGGCGACCTCA
-111-F	GAAATCAGGTATCCTGCGGTGTCCTGCGGTTACCAA
-111-R	GAACTTGGTAACCGCAGGACACCGCAGGATACCTGA
-128-F	GAAACATGGTCACCATAGGTCACCCTTGGCAACCAA
-128-R	GAACTTGGTTGCCAAGGGTGACCTATGGTGACCATG
-131-F	GAAAATGGGCCACCACGGGCGACCTCAGGTATCCTG
-131-R	GAACCAGGATACCTGAGGTCGCCCGTGGTGGCCCAT
-148-F	GAAATTGGCAACCAAAGGTGTCCTATGGCGGCCGGA
-148-R	GAACTCCGGCCGCCATAGGACACCTTTGGTTGCCAA
-151-F	GAAAAAGGGTGACCTATGGTGACCATGGGCCACCAC
-151-R	GAACGTGGTGGCCCATGGTCACCATAGGTCACCCTT
-168-F	GAAATATGGCGGCCGGAGGCTGCCATAGGACTCCGG
-168-R	GAACCCGGAGTCCTATGGCAGCCTCCGGCCGCCATA
-171-F	GAAAATAGGACACCTTTGGTTGCCAAGGGTGACCTA
-171-R	GAACTAGGTCACCCTTGGCAACCAAAGGTGTCCTAT
-198-F	GAAAGGAGTCCTATGGCAGCCTCCGGCCGCCATAGG
-198-R	GAACCCTATGGCGGCCGGAGGCTGCCATAGGACTCC
-208-F	GAAAGTAGGCCGGTCTCTCTAGAAGCGGCCGCGAAT
-208-R	GAACATTCGCGGCCGCTTCTAGAGAGACCGGCCTAC
-211-F	GAAATACGGTATCCACCGGAGTCCTATGGCAGCCTC
-211-R	GAACGAGGCTGCCATAGGACTCCGGTGGATACCGTA
tviB-NT1-F	GAAAAAATTCAACTGCTAGCGGCAATCCTACATAGC
tviB-NT1-R	GAACGCTATGTAGGATTGCCGCTAGCAGTTGAATTT
tviB-T1-F	GAAAGCTAGCAGTTGAATTTGGTAAACAGCGTTTGA
tviB-T1-R	GAACTCAAACGCTGTTTACCAAATTCAACTGCTAGC
tviC-NT1-F	GAAAGATAAAACCTGCAACACCTGTTATTAGCCAAG
tviC-NT1-R	GAACCTTGGCTAATAACAGGTGTTGCAGGTTTTATC
tviC-T1-F	GAAACAAAACTTGGCTAATAACAGGTGTTGCAGGTT

tviC-T1-R	GAACAACCTGCAACACCTGTTATTAGCCAAGTTTTG
ftsZ-NT1-F	GAAAGATGACTTTAATCACCGCGTCGTCTGAGTGAG
ftsZ-NT1-R	GAACCTCACTCAGACGACGCGGTGATTAAAGTCATC
ftsZ-T1-F	GAAAAATACAGACGCACAGGCGCTACGTAAATCTGG
ftsZ-T1-R	GAACCCAGATTTACGTAGCGCCTGTGCGTCTGTATT
pTarget-NT1-F	GAAAGTGATGTTAATGGGCACAAATTTTCTGTCAGT
pTarget-NT1-R	GAACACTGACAGAAAATTTGTGCCCATTAACATCAC
pTarget-NT2-F	GAAATTCAAGAGTGCCATGCCCGAAGGTTATGTACA
pTarget-NT2-R	GAACTGTACATAACCTTCGGGCATGGCACTCTTGAA
pTarget-NT3-F	GAAATTAACTTCAAAATTAGACACAACATTGAAGAT
pTarget-NT3-R	GAACATCTTCAATGTTGTGTCTAATTTTGAAGTTAA
pTarget-T1-F	GAAAATGCCCGAAGGTTATGTACAGGAAAGAACTAT
pTarget-T1-R	GAACATAGTTCTTTCCTGTACATAACCTTCGGGCAT
ribC-NT-72-F	GAAACAAGTGACCCAAACCATGATGGGGTTTATTGA
ribC-NT-72-R	GAACTCAATAAACCCCATCATGGTTTGGGTCACTTG
ribC-NT-87-F	GAAAATGATGGGGTTTATTGATACTGTCATGGCGGG
ribC-NT-87-R	GAACCCCGCCATGACAGTATCAATAAACCCCATCAT
ribC-T-171-F	GAAAAAAGACTGGTTCCCACTGCCACGGCAGCCATA
ribC-T-171-R	GAACTATGGCTGCCGTGGCAGTGGGAACCAGTCTTT
ribC-T-208-F	GAAAATAAGCAGGCCTTGTACAAACAAAATAGCCGG
ribC-T-208-R	GAACCCGGCTATTTTGTTTGTACAAGGCCTGCTTAT
bolA-NT-70-F	GAAACAACAAATCCGACAACATTTTGAATTCAACAA
bolA-NT-70-R	GAACTTGTTGAATTCAAAATGTTGTCGGATTTGTTG
bolA-T-171-F	GAAATCGTTGGAACTACACATTTAGGCAACTTGTCG
bolA-T-171-R	GAACCGACAAGTTGCCTAAATGTGTAGTTCCAACGA
bolA-T-188-F	GAAATAAAGGTCACAGAGCCTCGTTGGAACTACACA
bolA-T-188-R	GAACTGTGTAGTTCCAACGAGGCTCTGTGACCTTTA
bolA-T-196-F	GAAAGAAGCGCCTAAAGGTCACAGAGCCTCGTTGGA
bolA-T-196-R	GAACTCCAACGAGGCTCTGTGACCTTTAGGCGCTTC
crBFP-F	GAAAGTGGATAATCATCATTTTAAATGTACTAGCGA
crBFP-R	GAACTCGCTAGTACATTTAAAATGATGATTATCCAC

Table S3. Primers for qRT-PCR, PCR amplification and sequencing used in this study, related to STAR Methods.

Primer	Sequence
**Primers for qRT-PCR**	
gyrB-F	GGAACGACGGCTACCAAGA
gyrB-R	GTCAACGCACTACGGAAACC
omcA-F	CTGCCTAAAGCGATACCCGT
omcA-R	TGGAAAGCCCACGAAAGTGA
ribC-F	AGTTCAAGCCACCTGCGAG
ribC-R	CTCCAATCGCAAGTCCCTCA
bolA-F	GCTCGTCATCGCTTGGTGAA
bolA-R	GGCAATTAGGTGTTTTGGGCA
tviB-F	TTCAGCTACTTCAGGCGTGG
tviB-R	GTTTACCCAGGTGCGACTGA
tviC-F	TCTCGACTCGTTTCGCCATC
tviC-R	TGTTTGGTCGGCGTCAAGAT
ftsZ-F	TGCAACGGGTATAGGTGCTG
ftsZ-R	TTCCACAACGACTGGCTCTG
**Primers for PCR amplification and sequencing**	
Pae-crRNA-F	CACCTCGCTAACGGATTC
Pae-cr0-sacI-1F	CCGGTTGAGCTCGAATTCGCGGCCGC
Pae-cr0-1R	TTATTTGTTTCTTAGCTGCCGGCAGTGAACGCAACCATTAT
Pae-cr0-2F	GGCAGCTAAGAAACAAATAA
Pae-cr0-mluI-2R	GGCCGGACGCGTTGCGCGAGAAGATT

Table S4. PaeCascade-RpoD and crRNA cassette gene sequences used in this study, related to STAR Methods.

**PaeCascade-RpoD fusion gene sequence**
ttgacaattaatcatcggctcgtataatgtgtggaattgtgagcggataacaatttcacacaggaaacagccagtccgtttaggtgtttt
cacgagcacttcaccaacaaggaccatagcatatgacctctcccctcccaacgcctacgtggcaggagcttcgccagttcatcga
atccttcatccaggagcgcctccagggcaagctggacaaactccaacccgacgaagacgacaagcgccagacattgctggcc
acccaccggcgggaggcctggctggccgatgccgcccggcgggttggccagttgcagttggtgacccacacgctcaagccgat
ccatcccgacgcccgcggcagcaacctgcacagcctgccgcaagcacccggccaaccgggcctcgccggttcccatgagcta
ggtgacaggctggtcagcgatgtggtgggcaatgccgcggcgctggacgtattcaagtttctcagtctccagtatcagggtaaaaat
cttctgaactggctgacagaagacagtgccgaggcattacaggcgctgtccgataacgccgaacaggctcgcgaatggcggca
agcgttcatcggcatcacgaccgtcaaaggcgctcccgcgtcccacagcctggccaagcagttgtactttcccctgcccggttccg
gctaccacctgctagcaccgctgtttcccaccagtctggtgcatcacgtccacgctctgctccgcgaagcgcgcttcggcgacgcg
gccaaggcggcacgcgaagcgcgcagccgccaggagtcatggccccacggattcagcgagtaccccaacctggcgatccag
aagttcggcggtaccaagccgcagaacatcagtcagttgaacaacgagcgccgtggcgagaactggttgctgccatcgcttccg
ccgaactggcaaaggcagaatgtaaacgcgccgatgcggcactcttcggtcttcgagcatgacttcggaagaactcctgaagtat
cccgcctaacccgtaccctgcagcgttttcttgccaagacagtccataacaacctcgccatccgccagcggcgtgcccaattggtg
gcgcaaatctgcgacgaagccctgcaatacgccgcccgtctgcgcgaactggagcccggctggagcgcaaccccgggtgcc
aactgcatgacgcggagcagctctggctcgatccgttgcgcgcacagaccgatgagacgttcttgcagcgccgactacgaggtg
actggcctgccgaggtcggaaatcgcttcgccaactggctgaaccgggcggtcagcagcgacagtcagatactgggtagcccg
gaagccgcccaatggagccaggagctgagcaaggaactgacgatgttcaaggagatactcgaagatgagcgtgactgataat
aagatcccaactccataaggatccgggccctgtacaagatcctgtaaaacgacggccagtgaattcactcgagttctagaaataa
ttttgtttaactttaagaaggagatatagatcatgagcgtgactgatcccgaggcgctgctgttgctgccacgcctgtccatccagaac
gccaacgccatttccagccccttgacctggggtttcccttcgcccggcgctttcaccggattcgtccatgccttgcagcgtcgagtcgg
aatctccctcgatatcgaactggacggtgtcggcatcgtctgtcaccgcttcgaagcgcagatatcgcaaccggccggcaagcgc
accaaggtcttcaacctgacccgcaatccgctgaaccgcgacggctccaccgcagccatcgtcgaagaaggtcgcgcccatctg
gaggtcagtctgctgctcggagtgcatggcgatggtctggacgatcaccccgcacaggaaatcgccaggcaggtacaggagca
ggctggcgccatgcgcctcgccggtggcagcatcctgccctggtgcaatgagcgcttcccggctccgaacgccgaactgctgatg
ctgggtggcagcgacgaacagcggcgcaagaaccagcgccggcttacccgccgcctgctccccggcttcgccctggtgagtcg
cgaggcgttgttgcaacaacatctggaaaccttgcgcaccacgctccccgaagccaccacgctcgatgcactgctcgatctttgtc
gaatcaacttcgagcctcctgcaacctcgtccgaagaagaagcatcgccgcccgatgcagcctggcaagtgcgcgacaagccc
ggctggctggtgccgatcccggctggctacaacgccctgtcacccctgtacctccccggcgaagtgcgaaatgcccgtgatagag
aaacaccgctgcgcttcgtcgaaaacctcttcggtctgggcgaatggctcagcccccatcgcgtcgccgcgttatcggacctgcttt
ggtatcaccacgccgagcctgacaaagggctctaccgctggagcacaccccgtttcgtcgaacacgccatcgcataataataag
atcccaactccataaggatccgcgatcgcagcggataacaatttcacatcctgcaggactcgagttctagaaataattttgtttaactt
taagaaggagatatacatatgaaatcttctcaccatcaccatcaccatgaaaacctgtacttccaatccaatgcatccaagccaata
ctgagcaccgcttccgtcctcgccttcgaacgtaagctcgacccttccgacgcccttatgagcgctggcgcctgggcgcaacgcga
cgcctcgcaggaatggccggccgtgaccgtgcgcgagaagtccgtacgcggcaccatctccaaccgcctcaagaccaaggac
cgtgacccggccaagctggacgcctcgatccagtcgcccaacctgcagacggtggacgtggccaacctgccgagcgacgccg
acaccctcaaggtccgcttcaccctccgtgtgctcggcggcgccggaacaccgtctgcctgcaacgacgcggcctaccgtgaca
agctgctgcaaacggtcgccacctacgtgaacgatcagggcttcgccgagctggctcgtcgttatgcgcacaacctggccaacgc
ccgcttcctgtggcgcaaccgggtgggcgcggaagcggtggaagtccgtatcaaccatatccgccagggcgaggtggctcgcg
cctggcgcttcgacgccctggccatcggcttgcgcgacttcaaggccgacgccgaactggacgcgctcgccgaactgatcgcca

gcggcctctcaggcagtgggcatgtcctgctcgaagtggtcgccttcgcccgtatcggcgacggccaggaagtcttcccctcccag<br>gaactgatcctcgacaaaggcgacaagaaaggccagaagagcaagaccctgtacagcgtacgcgatgccgcggccatccac<br>tcgcagaagatcggcaatgccctgcgcaccatcgatacgtggtatcccgacgaagatggcctcggccccatcgccgtggagccc<br>tacggctccgtcacatcccagggcaaagcctatcgccagcccaagcagaagctggacttctatacgctgctcgacaactgggtac<br>tgcgcgacgaggcgcccgccgtggagcaacagcattatgtgatcgccaacctgatccgtggcggcgtgttcggtgaagccgaag<br>agaagggtggtggtggttctatgattagcatggatcataccccacaaagccaattaaaattattattagccaaaggcaaagaacaa<br>ggctatttaacctatgcagaagtgaatgatcatttacctgcagatatggtggatagtgatcaaattgaagatattattcaaatgattaat<br>gatatgggcattagagtgtttgaagaagcccctgatgcagatgatatgatgatgagtgaagataatactgatgaagatgcagcaga<br>agaagcagcagcagccttagccactgtggaaagtgaattaggcagaaccactgatcctgtgagaatgtatatgagagaaatggg<br>cactgtggaattattaactagagaaggtgaaattgtgattgccaaaagaattgaagaaggcattaatactgtgcaaagcagtgtgg<br>cagaatatccacaagccattgccatgattttagaacaatatgatcaatatgaagcagatgaattaagattaagtgatattattagtgg<br>ctttgtgaatcctgatgaagaggacttaggaccaactgccacccatattggcagtgaattaagtgaagaagatttagaggatgaag<br>atgatgaggaggatgatgaggatgaagatggtgatggtgatggtgatgatgatggcaataaaggccctgatcctgaagaagcaa<br>gagaaagatttagccaattaagaactgcctatgaaagtgccttaaaaattattgatgccaaaggcagagaacatcctgaaagcatt<br>caagcattatttgaaataggtgaaatttttaaggaatttagattagtgccaaaacaatttgacagattagtgaaaagcatgagaagc<br>atgatggatagagtgagagtgcaagaaagattattaatgaaattatgtgtggaacaagccaaaatgccaaaaaaaaattttgtga<br>aattttttactggcaatgaaaccaatttagattggtttgaagcagaaaaaactagcaataaaccatatgcagaaggcttaagaatgg<br>tggaagaagatgtgcaaagatgtagaagcaaattagcagccattgaagaagaaactggcttagtgattgcagccattaaagatat<br>taatagaagaatgagcattggtgaagccaaggcaagaagagccaaaaaagaaatggtggaagccaatttaagattagtgatta<br>gcattgccaaaaaatataccaatagaggcttacaatttttagatttaattcaagaaggcaatattggcttaatgaaagcagtggataa<br>atttgaatatagaagaggctataaatttagcacctatgccacctggtggattagacaagccattactagaagcattgcagatcaagc<br>aagaaccattagaattcctgtgcatatgattgaaaccattaataaattaaatagaattagcagacaaatgttacaagaaatgggca<br>gagaaccaagccctgaagaattagcagaaagaatgatgatgcctgaagataaaattagaaaagtgttaaaaattgccaaagaa<br>ccaattagcatggaaaccccaattggtgatgatgaagatagtcacttaggtgattttattgaagataccaccttagaattaccattaga<br>tagtgccactagtgaaagcttaaaaagtgccacccatgaagtgttagctggcttaactgcaagagaagccaaagtgttaagaatg<br>agatttggcattgatatgaatactgatcataccttagaagaagtgggcaaacagtttgatgtgactagagaaagaattagacaaatt<br>gaagcaaaggctctgagaaaattaagacatccaagcagaagtgaaattttaaaaagctttttagatgaataataataacattggaa<br>gtggataacggatccgcgatcgcggcgcgcccgccagggttttcccagtcgcggccgccctgcaggactcgagttctagaaata<br>attttgtttaactttaagaaggagatatagatcatggaccactacctcgacattcgcttgcgaccggacccggaatttcccccggcgc<br>aactcatgagcgtgctcttcggcaagctccaccaggccctggtggcacagggcggggacaggatcggcgtgagcttccccgacc<br>tcgacgaaagccgctcccggctgggcgagcgcctgcgcattcatgcctcggcggacgaccttcgtgccctgctcgcccggccctg<br>gctggaagggttgcgggaccatctgcaattcggagaaccggcagtcgtgcctcaccccacaccgtaccgtcaggtcagtcgggtt<br>caggcgaaaagcaatccggaacgcctgcggcggcggctcatgcgccggcacgatctgagtgaggaggaggctcggaaacg<br>cattcccgatacggtcgcgagagccttggacctgcccttcgtcacgctacgcagccagagcaccggacagcacttccgtctcttcat<br>ccgccacgggccgttgcaggtgacggcagaggaaggaggattcacctgttacgggttgagcaaaggaggtttcgttccctggttct<br>gagctagcggctcgagcaaataaaacgaaaggctcagtcgaaagactgggcctttcgttttatctgttgtttgtcggtgaacgctctc<br>ctgagtaggacaaat
**crRNA cassette gene sequence** a)
taacaccgtgcgtgttgactattttacctctggcggtgataatggttgcgttcactgccgtataggcagctaagaaaa**gagacc**aaa<br>**ggtctc**ggttcactgccgtataggcagctaagaaacaaataaaacgaaaggctcagtcgaaagactgggcctttcgttttat

[a] BsaI recognition sites are bolded.

Chen, Y., Fang, L., Ying, X., Cheng, M., Wang, L., Sun, P., Zhang, Z., Shi, L., Cao, Y., and Song, H. (2022). Development of Whole Genome-Scale Base Editing Toolbox to Promote Efficiency of

Extracellular Electron Transfer in Shewanella oneidensis MR-1. Advanced biology, e2101296.
Chowdhury, S., Carter, J., Rollins, M.F., Golden, S.M., Jackson, R.N., Hoffmann, C., Nosaka, L., Bondy-Denomy, J., Maxwell, K.L., Davidson, A.R., *et al.* (2017). Structure Reveals Mechanisms of Viral Suppressors that Intercept a CRISPR RNA-Guided Surveillance Complex. Cell *169*, 47-57 e11.
